# Characterization of Linoleate 10-Hydratase of *Lactobacillus plantarum* and Novel Antifungal Metabolites

**DOI:** 10.3389/fmicb.2016.01561

**Published:** 2016-10-04

**Authors:** Yuan Y. Chen, Nuan Y. Liang, Jonathan M. Curtis, Michael G. Gänzle

**Affiliations:** ^1^Department of Agricultural, Food and Nutritional Science, University of Alberta, EdmontonAB, Canada; ^2^College of Bioengineering and Food Science, Hubei University of TechnologyWuhan, China

**Keywords:** linoleate 10-hydratase, cell membrane fluidity, cell surface hydrophobicity, 13-hydroxy-9-octadecenoic acid, antifungal activity

## Abstract

Lactobacilli convert linoleic acid to the antifungal compound 10-hydroxy-12-octadecenoic acid (10-HOE) by linoleate 10-hydratase (10-LAH). However, the effect of this conversion on cellular membrane physiology and properties of the cell surface have not been demonstrated. Moreover, *Lactobacillus plantarum* produces 13-hydroxy-9-octadecenoic acid (13-HOE) in addition to 10-HOE, but the antifungal activity of 13-HOE was unknown. Phylogenetic analyses conducted in this study did not differentiate between 10-LAH and linoleate 13-hydratase (13-LAH). Thus, linoleate hydratases (LAHs) must be characterized through their differences in their activities of linoleate conversion. Four genes encoding putative LAHs from lactobacilli were cloned, heterologous expressed, purified and identified as FAD-dependent 10-LAH. The unsaturated fatty acid substrates stimulated the growth of lactobacilli. We also investigated the role of 10-LAH in ethanol tolerance, membrane fluidity and hydrophobicity of cell surfaces in lactobacilli by disruption of *lah*. Compared with the *L. plantarum lah* deficient strain, 10-LAH in wild-type strain did not exert effect on cell survival and membrane fluidity under ethanol stress, but influenced the cell surface hydrophobicity. Moreover, deletion of 10-LAH in *L. plantarum* facilitated purification of 13-HOE and demonstration of its antifungal activity against *Penicillium roqueforti* and *Aspergillus niger*.

## Introduction

Antifungal metabolites of lactic acid bacteria have potential for applications as antifungal preservatives in cereal products, and in silage (Magnusson et al., 2003; Oliveira et al., 2014). Several hydroxy fatty acids have antifungal activity (Hou and Forman Iii, 2000; Hou, 2008) and antifungal 3-hydroxy fatty acids of C_10_ to C_14_ chain lengths are formed by *Lactobacillus plantarum* MiLAB 14 (Sjögren et al., 2003). *Lactobacillus hammesii* accumulates 10-HOE, an antifungal compound that increased the mold-free shelf life of bread (Black et al., 2013a,b). The biosynthesis of antifungal hydroxy fatty acids and the application of hydroxy fatty acids in food are dependent on knowledge of enzymes involved in microbial fatty acid-hydroxylation (Kim and Oh, 2013).

Linoleate hydratase (LAH) activity was first characterized in *Streptococcus pyogenes*; the hydratase was previously described as myosin cross-reactive antigen (Kil et al., 1994). The FAD containing LAH in *S. pyogenes* hydrates the *cis*-9 and *cis*-12 double bonds of C_16_ and C_18_ fatty acids to produce 10-hydroxy and 10,13-dihydroxy fatty acids (Volkov et al., 2010). Remarkably, the LAHs of bifidobacteria, lactobacilli and *Nocardia* spp. exclusively hydrate *cis*-9 double bond (Koritala and Bagby, 1992; Rosberg-Cody et al., 2011; Yang et al., 2013). The crystal structure of the LAH from *Lactobacillus acidophilus* provided the structural basis for the selective substrate recognition of linoleate 10-hydratase (Volkov et al., 2013). A second LAH in *L. acidophilus* hydrates the *cis*-12 double bond to produce 13-hydroxy fatty acid (Kim et al., 2015; Park et al., 2015).

The physiological and ecological function of LAHs -have been investigated. The LAH/myosin-cross reactive antigen of *S. pyogenes* mediated adherence to human keratinocytes; LAH was also suggested to detoxify linoleic acid by conversion to a hydroxyl-product with lower antibacterial activity (Volkov et al., 2010). Similarly, a *L. acidophilus* mutant with truncated LAH exhibited a decreased adherence to intestinal epithelial cells, and was more sensitive to stresses (O’Flaherty and Klaenhammer, 2010). Heterologous expression of a LAH of *Bifidobacterium breve* in *Lactococcus lactis* increased resistance to heat and solvent stresses (Rosberg-Cody et al., 2011). Linoleate 10-hydratase is predicted to be a membrane-associated protein with one trans-membrane helix (Kil et al., 1994; Rosberg-Cody et al., 2011), and hydration of linoleic acid occurs in the cell periphery (Kishino et al., 2011). However, the effect of linoleic acid hydratase on properties of cell membranes and the cell surface have not been demonstrated. Moreover, 10-HOE has antifungal activity (Black et al., 2013b) but a corresponding activity of alternative hydration products of fatty acids remains unknown.

This study aimed to characterize linoleic acid hydratases in *L. plantarum, Lactobacillus reuteri, L. hammesii*, and *Lactobacillus spicheri*. Strain selection included strains which produce only 10-HOE and strains that produce 10-HOE and 13-HOE (Black et al., 2013b). Four enzymes were characterized after heterologous expression in *Escherichia coli*. The physiological function of the linoleate 10-hydratase of *L. plantarum* was studied in more detail by comparison of the ethanol resistance and cell surface properties of *L. plantarum* TMW1.460 and its linoleate 10-hydratase deficient mutant *L. plantarum* TMA1.460Δ*lah*. The antifungal activity of 13-HOE was compared to the activities of other hydroxy fatty acids.

## Materials and Methods

### Bacterial Strains and Fermentation

*Lactobacillus reuteri* LTH2584, *L. plantarum* TMW1.460, *L. hammesii* DSM16381, *L. spicheri* Lp38 and *Lactobacillus sanfranciscensis* ATCC 27651 were anaerobically cultivated at 37°C (*L. reuteri*) or 30°C (all other strains) in modified De Man Rogosa Sharpe (mMRS) medium containing 0.1% Tween 80 (mMRS-Tween 80; Zhang et al., 2010). mMRS-Tween 20 was prepared by replacing Tween 80- with an equal weight of Tween 20. *E. coli* DH5α (New England Biolabs) served as a host for plasmids in the cloning procedures, and *E. coli* BL21 Star (DE3) (ThermoFisher Scientific) was used for protein overexpression. *E. coli* strains were cultivated in Luria-Bertani (LB) medium (BD, Mississauga, CA, USA) with agitation at 200 rpm and 37°C. Antibiotic-resistant *E. coli* carrying plasmid pET-28a(+), pUC19 or pJRS233 were cultured in media containing 50 mg/L kanamycin, 50 mg/L ampicillin, or 500 mg/L erythromycin, respectively. Erythromycin-resistant *L. plantarum* was grown in presence of 5 mg/L erythromycin. *Aspergillus niger* FUA5001 and *Penicillium roqueforti* FUA5005 were grown at 25°C for 72 h on malt extract agar.

Linoleic acid metabolism of lactobacilli was analyzed after incubation in mMRS-Tween80 broth supplied with 5% inoculum and 4 g/L linoleic acid anaerobically for 4 days. Lipids were isolated by addition of one volume of 85:15 (vol/vol) chloroform-methanol to cultures prior to incubation 4°C overnight; cultures were then extracted twice with additional two volumes of chloroform–methanol (85:15, vol/vol). The organic solvent was evaporated under reduced pressure and the dry residue stored at -20°C under nitrogen.

### DNA Manipulations

Genomic DNA was isolated using the Blood & Tissue Kit (Qiagen, Hilden, Germany). Plasmid DNA from *E. coli* was extracted with a QIAprep Spin Miniprep kit (Qiagen). PCR primers used in this study were synthesized by Integrated DNA Technologies (San Diego, CA, USA). The Taq DNA polymerase was purchased from TaKaRa Bio (Shiga, Japan). T4 DNA ligase and restriction enzymes were obtained from Thermo Scientific (Mississauga, CA, USA). PCR products were purified by using the DNA gel extraction kit (Qiagen). DNA sequencing was performed by Macrogen (Rockville, MD, USA).

### Sequence and Phylogenetic Analysis of Linoleate Hydratases in Lactobacilli

Genes of putative LAHs in *L. spicheri* Lp38, *L. reuteri* LTH2584 and *L. plantarum* TMW1.460 were amplified with primers that are specific to LAHs in genome-sequenced strains of these species (**Table 1**). To identify the LAH in *L. hammesii, lah* sequences from the closely related *L. spicheri* and *L. brevis* were aligned and specific primers targeting conserved sequences upstream and downstream of *lah* coding sequences were designed (**Table 1**). The four *lah* genes were sequenced by service of Macrogen (Rockville, MD, USA).

**Table 1:** Primers used in this study.

Primers [forward (F); reverse (R)]	Sequence (5′–3′)	Restriction site^a^
DSM16381 sequencing, F1	5′-TACGGAGGTGTTTTTTGATGGT-3′	—
DSM16381 sequencing, R1	5′-CGTAAATTCATAAATCATTTGGTGCATGTA-3′	—
DSM16381 sequencing, F2	5′-TACATGCACCAAATGATTTATGAATTTACG-3′	—
DSM16381 sequencing, R2	5′-TACTTCGTCTTAGGTGACCA-3′	—
LTH2584 cloning, F	5′-CGCCATATGTACTATTCAAACGGAAATTATG-3′	NdeI
LTH2584 cloning, R	5′-ATTTGCGGCCGCTTAAAGTAAATGTTGTTCTTCCATT-3′	NotI
TMW1.460 cloning, F	5′-CCGGAATTCATGGTTAAAAGTAAAGCAATTATGA-3′	EcoRI
TMW1.460 cloning, R	5′-ATTTGCGGCCGCTTAATCAAACATCTTCTTAGTTGC-3′	NotI
DSM16381 cloning, F	5′-CGCGGATCCATGGTTAAAACAAAAGCAGTAATG-3′	BamHI
DSM16381 cloning, R	5′-CCCAAGCTTTTAGCTAAACATCCGCTTCGTTGC-3′	HindIII
Lp38 cloning, F	5′-CCGGAATTCATGGTTAAGACAAAAGCTGTAATG-3′	EcoRI
Lp38 cloning, R	5′-ATAGTTTAGCGGCCGCGTTAACTAAACATTTTCTTCGTTGCC-3′	NotI
*Lah*-upstream A, F	5′-AACTGCAGGCCTAAAACGAGCTAAACGAC-3′	PstI
*Lah*-upstream A, R	5′-ACATGCATGCCCCGGCACCAATCATAATTGCTTTAC-3′	SphI
*Lah*-downstream B, F	5′-ACATGCATGCAAGAAGATGTTTGATTAATTAAA-3′	SphI
*Lah*-downstream B, R	5′-CCCAAGCTTATGAAAAAATTAACATCAGTCG-3′	HindIII

*^a^Restriction sites are underlined.*

The phylogenetic analysis of LAHs included the type strains of the 24 groups of the -genus *Lactobacillus sensu lato* (Zheng et al., 2015) and the genera *Weissella, Leuconostoc*, and *Oenococcus*. Sequences of biochemically characterized linoleate 10-hydratase and linoleate 13-hydratase from *Lactobacillus* spp., *Bifidobacterium* spp., and *Streptococcus* spp. were included in the phylogenetic analysis (Volkov et al., 2010; Rosberg-Cody et al., 2011; Yang et al., 2013; Kim et al., 2015). Protein sequences of LAH were retrieved from GenBank^1^, using NCBI BLAST analysis with organism specific search. Protein phylogenetic tree was built in MEGA7.

### Cloning and Heterologous Expression of Linoleate Hydratases of Lactobacilli

Coding regions of the four *lah* were amplified from genomic DNA of the respective strains with primers listed in **Table 1**. Amplicons were purified and cloned into pGEM-T Easy vector (Promega, Madison, WI, USA). The *lah* fragments in recombinant pGEM-T vectors were digested with restriction endonucleases (**Table 1**), purified and ligated into expression vector pET-28a(+) (Novagen, Toronto, ON, Canada), yielding the respective constructs pET28a/LAH for each strain. Recombinant plasmids were introduced into chemically competent *E. coli* BL21 Star (DE3) (Thermo Fisher Scientific) and transformants were plated on LB agar containing 50 mg/L kanamycin. The gene cloning was verified by PCR amplification and sequencing.

Four recombinant *E. coli* BL21 (DE3) strains were grown to an optical density (OD) at 600 nm of 0.6. Protein expression was induced by the addition of isopropyl-β-D-thiogalactopyranoside (IPTG) to a final concentration of 1.0 mM, followed by incubation for 4 h and harvesting of cells by centrifugation at 4°C.

### Purification of Linoleate Hydratases

Overexpressed LAHs were present mainly as inclusion bodies. Solubilization and refolding of inclusion bodies were carried out with the protein refolding kit (Novagen) according to the manufacturer’s instructions. The refolded proteins were concentrated by using a 10 KDa Amicon Ultra-15 centrifugal filter Unit (Millipore, Germany).

After concentration, the His-tagged LAHs were purified by Ni-NTA spin columns (Qiagen). The purified LAHs were finally dialyzed against 50 mM 4-morpholineethanesulfonic acid (MES) buffer (pH 6.1) (Sigma-Aldrich) overnight at 4°C. The purified enzymes were assessed by SDS-PAGE and staining with Coomassie blue. FAD was added to the purified enzymes at a final concentration of 0.2 mM and incubated at 4°C for 24 h (Joo et al., 2012).

### Enzymatic Activity Assay and Fatty Acid Analysis

To determine the enzymatic activity, 4.5 mg linoleic acid and 25 μg purified LAH were incubated in 1 ml of 50 mM MES buffer (pH 6.1) containing 50 mM NaCl, 2% ethanol and 10% glycerol at 25°C for 3 h. Fatty acids were extracted following the procedure described above. The organic phase of the extracts was collected and analyzed with LC-APPI-MS as described (Black et al., 2013a,b).

### Construction of *L. plantarum* TMW1.460Δ*lah* by Double-Crossover Mutagenesis

Gene disruption of *lah* in *L. plantarum* TMW1.460 was achieved by an in-frame, unmarked deletion (Su et al., 2011). The approximately 900 bp 5′-flanking regions (fragment A) and 1000 bp 3′-flanking regions (fragment B) of *lah* were amplified by PCR with primers listed in **Table 1**. The fragment A was digested with PstI, SphI, and fragment B was digested with SphI, HindIII. The resulting fragments were purified and sequentially ligated into vector pUC19 to generate pUC19/AB. The AB fragment in pUC19/AB was confirmed by sequencing, and sub-cloned into the PstI and HindIII restriction sites of pJRS233 to create pJRS233/Δ*lah*. Recombinant plasmids were transformed into electrocompetent *L. plantarum* TMW1.460 at 12.5 kV/cm, 25 μF, and 200 Ω. The cells were grown in mMRS-Tween80 broth containing 5 mg/l erythromycin at 42–44°C for 80 generations to select for single-crossover mutants. Several colonies were isolated, cultured in mMRS-Tween80 broth for approximately 100 generations, and plated on mMRS-Tween80 agar at 30°C. The colonies were replica plated on mMRS-Tween80-erythromycin agar to identify erythromycin-sensitive double-crossover mutants. Gene replacement was confirmed by PCR amplification and sequencing. The phenotype was determined by LC-APPI-MS analysis of culture supernatant of *L. plantarum* TMW1.460Δ*lah* grown in mMRS-Tween80 supplemented with 4 g/L linoleic acid.

### Determination of Ethanol Resistance

Ethanol tolerance of *L. plantarum* TMW1.460 and TMW1.460Δ*lah* was carried out with strains grown in mMRS-Tween 80, or mMRS-Tween 20. Ethanol was added as indicated and media were sterilized by filtration after addition of ethanol. Stationary phase cells were harvested by centrifugation, and resuspended in the same volume of mMRS-Tween 80 or -Tween 20 containing 20% ethanol. A 100 μl aliquot of cell suspension was analyzed as untreated control. The samples were incubated in 30°C and aliquots were removed in 1.5 h intervals and serially diluted in 0.85% NaCl. The appropriate dilutions were surface plated in duplicate on respective mMRS-Tween80 or -Tween20 agar and incubated at 30°C for 24 h. Ethanol resistance was determined in three independent experiments.

### Determination of the Membrane Fluidity under Ethanol Stress

To investigate the membrane fluidity, LAURDAN (6-dodecanoyl-2-dimethylaminonaphthalene) (Thermo Fisher Scientific) was employed to measure generalized polarization (GP) value. *L. plantarum* TMW1.460 and TMW1.460Δ*lah* were cultivated at 30°C for 20 h in mMRS-Tween80 or mMRS-Tween 20. The membrane fluidity of the cells influenced by ethanol at the concentration ranging from 0 to 16% was assessed as described (Molina-Höppner et al., 2004). The effect was determined in three independent experiments.

### Physicochemical Properties of Cells Surface

Cell surface hydrophobicity was assessed by quantification of microbial adhesion to solvents (MATS; Kankaanpää et al., 2004). Solvents used were: chloroform (polar and electron acceptor) and tetradecane (non-polar), ethyl acetate (polar and electron donor) and octane (non-polar). The MATS is based on the comparison between microbial cell affinity to a polar and non-polar solvent within a couple that pose similar van der Waals surface tension components.

*Lactobacillus plantarum* TMW1.460 and TMW1.460Δ*lah* were grown in mMRS-Tween 20, or mMRS without Tween but supplemented with 1 g/L oleic acid or linoleic acid. Cells were harvested by centrifugation, washed twice and resuspended in 150 mM NaCl to a final cell concentration of 10^8^ CFU/ml. A 1 ml aliquot was removed as untreated control (A_0_). A 2.4 ml aliquot of cell suspension was mixed with 0.4 ml of solvent by vortexing for 60 s, respectively. The emulsified mixture was allowed to stand for 20 min to ensure the complete separation of the two phases. A sample (1 ml) was carefully taken from the aqueous phase (A). The optical cell density of sample A_0_ and A was measured at 600 nm. The microbial adhesion percentage to each solvent was calculated with the equation: percent affinity = 100 × [1-(A/A_0_)]. Each measurement was determined in three independent experiments.

### Extraction and Purification of 13-HOE and 10-HOE

Extraction and purification of 13-HOE and 10-HOE was based on a protocol developed for 10-HOE (Black et al., 2013b). Fermentation of *L. hammesii* or *L. plantarum* TMW1.460Δ*lah* in mMRS-Tween 80 with 4 g/L linoleic acid was conducted for production of 10-HOE or 13-HOE, respectively. The crude extraction of cellular lipids was performed as described above. Subsequently, the purification of 10-HOE or 13-HOE was based on a protocol described previously (Black et al., 2013b). The fermentation of *L. hammesii* or *L. plantarum* TMW1.460Δ*lah* in mMRS-Tween 80 with 4 g/L linoleic acid and the crude extraction of fatty acid mixtures were performed as described above. For further purification, 25 mg of sample dissolved in chloroform was applied to a 500-mg Sep-Pak silica cartridge (Waters, Ltd, Mississauga, ON, Canada) previously equilibrated with 6 ml chloroform. The cartridge was successively washed with the following gradient of isopropanol in chloroform: 35 ml of chloroform, followed by 18 ml of 1, 5, 10, and 50% (vol/vol) isopropanol in chloroform. Eluates were collected and concentrated to dryness under nitrogen. The dry residues were dissolved in chloroform for analysis by LC-APPI-MS to identity of the product, and the removal of contaminating lipids.

### Determination of the Antimicrobial Activity of Fatty Acids

The minimum inhibitory concentration (MIC) was determined to assess the toxicity of linoleic acid to lactobacilli, and to determine the antifungal activity of 10-HOE, 13-HOE, coriolic acid, ricinoleic acid, and linoleic acid. Lipids dissolved in ethanol were serially diluted twofold in mMRS-Tween80 using 96-well microtiter plates. Ethanol in the samples was evaporated under a sterile laminar flow hood before inoculation with indicator strains. Stationary phase cells of lactobacilli were harvested by centrifugation, washed twice in mMRS-Tween80 and diluted to approximately 10^7^ CFU/ml in the same medium. The plates were incubated at 30°C.

For evaluation of the antifungal activity, lipids diluted with serial twofold dilutions. Conidiospores of *A. niger* and *P. roqueforti* were prepared as reported (Black et al., 2013b). The plate was inoculated with *A. niger* as an indicator organism and incubated at 25°C for 2 days, while the plate inoculated with *P. roqueforti* was incubated for 3 days. Fungal growth without addition of lipids served as the positive control and media alone as the negative control. The MIC was defined as the lowest concentration of lipids that inhibited the growth of fungi when growth was visible in the positive control. MIC values were determined by six independent experiments.

### Statistical Analysis

Data analysis was conducted with R 3.1.2 (R Core Team, 2014). Significant differences in the assessment of cell survival and membrane fluidity under ethanol stress were determined by one-way analysis (ANOVA). Significance was assessed at a 5% probability of error (*P* < 0.05).

### Accession Numbers

The sequences of linoleate 10-hydratase in *L. reuteri, L. plantarum, L. hammesii*, and *L. spicheri* were deposited in GenBank with accession numbers KX827285, KX827286, KX827287, KX827288, respectively.

## Results

### Identification of the Products of Linoleate Conversion by Lactobacilli

**FIGURE 1:**
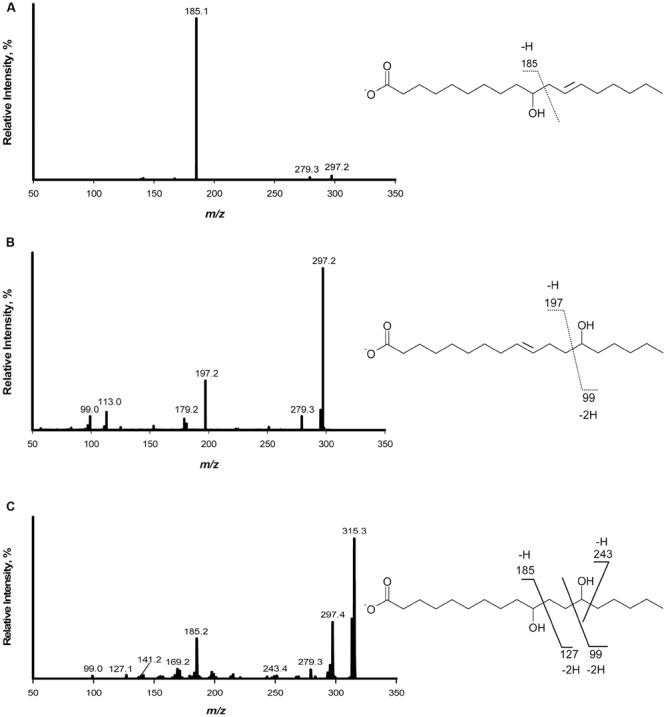
**Fragmentation pattern and APPI-MS/MS spectra of hydroxy fatty acids produced when linoleic acid was used as substrate. (A)** Mass spectrum of 10-hydroxy-12- octadecenoic acid; **(B)** Mass spectrum of 13-hydroxy-9-octadecenoic acid; **(C)** Mass spectrum of 10,13-dihydroxy octadecanoic acid.

The products of linoleic acid conversion by five lactobacilli were analyzed by negative ion LC/APPI-MS/MS (**Table 2**). MS/MS spectra of the products confirmed the position of hydroxyl groups (**Figure 1**; Black et al., 2013a). The strains of lactobacilli differed in with respect to their conversion of linoleic acid to hydroxy fatty acids (**Table 2**). *L. reuteri, L. hammesii*, and *L. spicheri* produced 10-HOE only, while *L. plantarum* produced 10-HOE, 13-HOE and 10,13-dihydroxy octadecanoic acid. *L. plantarum* TMW1.460Δ*lah* produced 13-HOE but not 10-HOE or 10,13-dihydroxy octadecanoic acid, demonstrating that their formation by *L. plantarum* TMW1.460 is attributable to a dedicated linoleate 13-hydratase acting on linoleic acid and 10-HOE, respectively. *L. sanfranciscensis* did not convert linoleic acid.

**Table 2:** Comparison of products obtained from strain fermentation and enzymatic reaction with linoleic acid as substrate.

Strain	Products of fermentation	Products of LAH	Fragmentation ions (*m/z*)
*L. reuteri* LTH2584	10-HOE^a^	10-HOE	297[M-H]^-^, 185
*L. hammesii* DSM16381	10-HOE	10-HOE	297[M-H]^-^, 185
*L. spicheri* LS38	10-HOE	10-HOE	297 [M-H]^-^, 185
*L. plantarum* TMW1.460	10-HOE, 13-HOE^a^ and 10,13-HOA^a^	10-HOE	297 [M-H]^-^, 185 297 [M-H]^-^, 99, 197 315 [M-H]^-^, 99, 127, 185, 243
*L. plantarum* TMW1.460 Δ*lah*	13-HOE	—	297 [M-H]^-^, 99, 197
*L. sanfranciscensis* ATCC 27651	No products	No products	—

*^a^10-HOE, 10-hydroxy-12-octadecenoic acid; 13-HOE, 13-hydroxy-9-octade cenoic acid; 10,13-HOA, 10,13-dihydroxy octadecanoic acid.*

### Phylogenetic Analysis of Linoleate Hydratase

**FIGURE 2:**
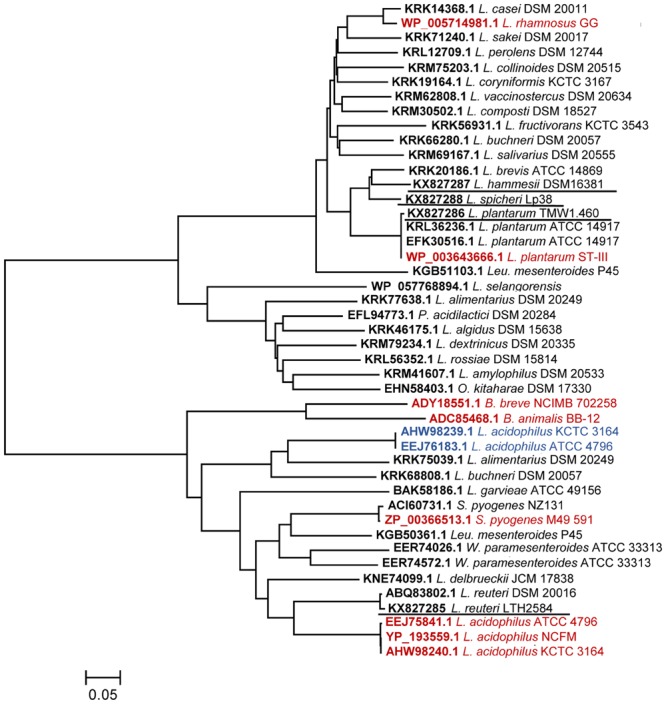
**Phylogenetic tree of linoleate hydratases (LAHs).** The evolutionary relationships are shown with scale bar line which represents an evolutionary distance of 0.05. Linoleate 10-hydratases reported in the literature are highlighted in red, linoleate 13-hydratases are highlighted in blue, linoleate 10-hydratases that were characterized in this study are underlined.

Phylogenetic relationships of putative LAHs from lactobacilli were compared to the corresponding enzymes of the 24 type strains in the genus *Lactobacillus*, and type strains from the genera *Pediococcus, Weissella, Leuconostoc*, and *Oenococcus* (**Figure 2**). All four hydratases from lactobacilli that were investigated in this study belonged to myosin cross reactive antigen family. *L. sanfranciscensis* harbored no LAH. The topology of the protein tree did not conform to the evolutionary relationship of the organisms (Zheng et al., 2015). The tree displayed two clusters but the two clusters do not differentiate between linoleate 10-hydratases and linoleate 13-hydratases (**Figure 2**).

### Characterization of Linoleate 10-Hydratase

**FIGURE 3:**
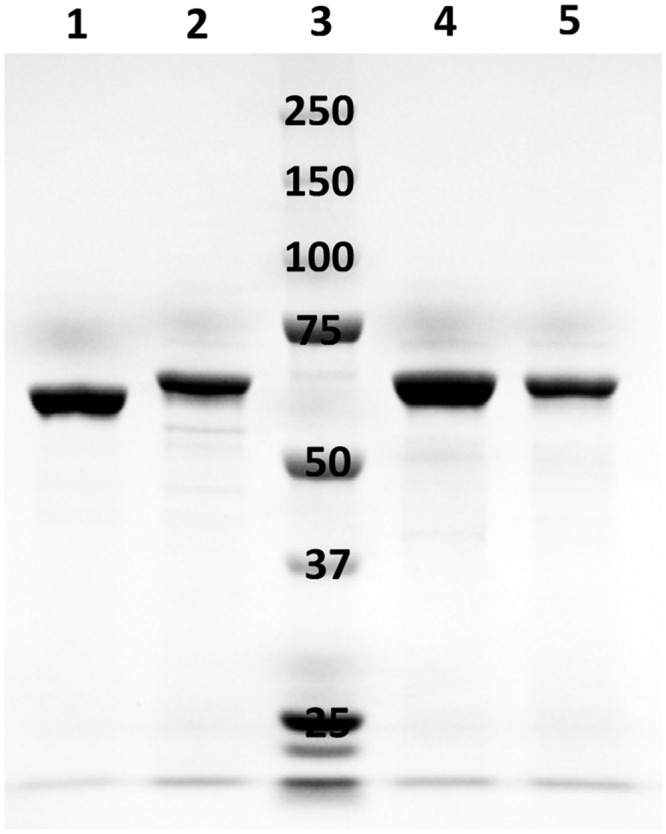
**SDS-PAGE analysis of purified LAHs expressed from respective recombinant *Escherichia coli* BL21 (DE3) cells.** Lane 1, LAH of *Lactobacillus reuteri*; lane 2, LAH of *Lactobacillus plantarum*; lane 3, molecular mass marker proteins (250, 150, 100, 75, 50, 37, and 25 kDa); lane 4, LAH of *Lactobacillus hammesii*; lane 5, LAH of *Lactobacillus spicheri*.

Sequence analysis did not distinguish 10-hydratases from 13-hydratases, therefore, LAHs from four strains of lactobacilli were characterized biochemically after heterologous expression in *E. coli* and purification by affinity chromatography. A single band was observed by SDS-PAGE analysis after purification of the four enzymes (**Figure 3**); this band was absent in crude cellular extracts of *E. coli* strains prior to induction (data not shown). The genes from *L. reuteri, L. plantarum, L. hammesii*, and *L. spicheri* encoded a protein of 590, 564, 564, 564 amino acids, respectively, matching the size of the major band observed by SDS-PAGE analysis (**Figure 3**). The addition of cofactor FAD to apoenzyme was essential for activity. LC-APPI-MS analysis revealed that all four recombinant proteins produced 10-HOE from linoleic acid, as demonstrated by the fragment ion at *m*/*z* 185.1 in the MS/MS spectra (**Table 2**). Hence, all four recombinant proteins were shown to be 10-hydratases. The substrate specificity of the linoleate 10-hydratase of *L. plantarum* TMW1.460 confirmed that formation of 13-HOE and 10,13-dihydroxy octadecanoic acid by this strain is likely attributable to a second and dedicated linoleate 13-hydratase.

### Most Lactobacilli Require Oleic Acid or Linoleic Acid for Growth

Tween 80 is a derivative of oleate and a component of mMRS (Johnsson et al., 1995; Gänzle et al., 2000). mMRS-Tween20 did not support the growth of *L. reuteri, L. hammesii*, and *L. sanfranciscensis* but did support the growth of *L. plantarum* and its Δ*lah* mutant (data not shown). However, unsaturated fatty acids (UFAs) may also exhibit antibacterial activity (Desbois and Smith, 2010). Therefore, the MICs of oleic and linoleic acid to lactobacilli including *L. plantarum* TMW1.460Δ*lah* were assessed. The MICs of oleic and linoleic acids were >8 g/L for all strains, which indicates a high tolerance toward oleic and linoleic acid. *L. sanfranciscensis* showed less tolerance and its growth was inhibited by 1 g/L oleic acid and 0.5 g/L linoleic acid.

### The Effect of *lah* on Stress Tolerance in *L. plantarum*

**FIGURE 4:**
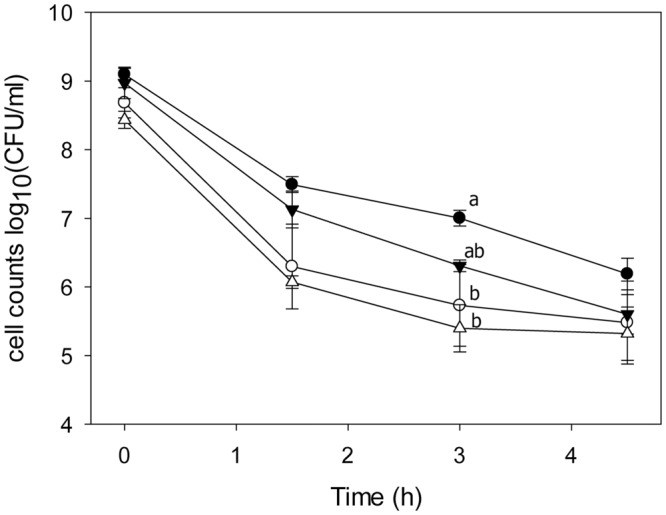
**Survival of *L. plantarum* and its *lah* deficient derivative under 20% ethanol treatment.** *L. plantarum* TMW1.460 was incubated in mMRS-Tween80 (closed circles) or mMRS-Tween20 (open circles); *L. plantarum* TMW1.460Δ*lah* was grown in mMRS-Tween80 (closed triangle) or mMRS-Tween 20 (open triangle) during treatment. Values obtained at the same treatment time that do not share common superscripts are significantly different (*P* < 0.05). Data represent mean ± standard deviation of three independent experiments with duplicate determinations of cell counts.

To investigate the effect of hydroxy fatty acids or 10-LAH itself on ethanol resistance, the survival of *L. plantarum* TMW1.460 and TMW1.460Δ*lah* was assessed in mMRS-Tween80 and mMRS-Tween20 containing 20% ethanol. The ethanol tolerance of *L. plantarum* TMW1.460 and TMW1.460Δ*lah* did not differ (**Figure 4**). However, the presence of Tween 80 in the growth medium enhanced bacterial survival, especially for *L. plantarum* TMW1.460 (**Figure 4**).

### The Effect of *lah* on Ethanol-Dependent Membrane Fluidity in *L. plantarum*

**FIGURE 5:**
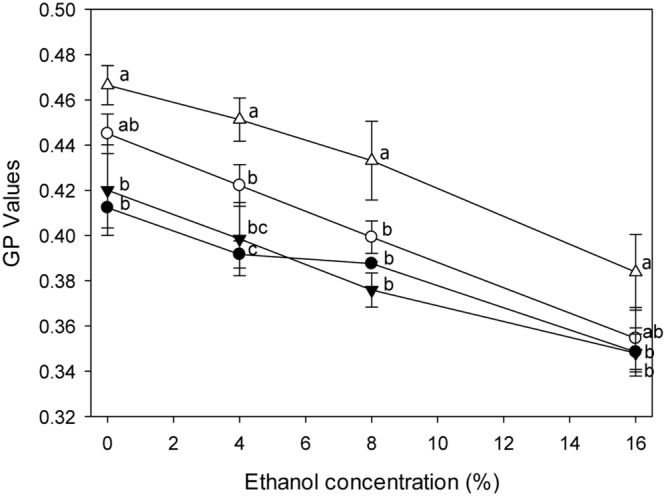
**General polarization (GP) values of *L. plantarum* and its Δ*lah* derivative stained with Laurdan under ethanol stress.** *L. plantarum* TMW1.460 was cultivated in mMRS-Tween80 (closed circles) or mMRS-Tween 20 (open circles) prior to staining; *L. plantarum* TMW1.460Δ*lah* was cultivated in mMRS-Tween80 (closed triangle) or mMRS-Tween 20 (open triangle) prior to staining. Values obtained at the same treatment time that do not share common superscripts are significantly different (*P* < 0.05). Data represent mean ± standard deviation of three independent experiments with duplicate determinations of cell counts.

The ethanol-dependent membrane phase behavior of strains grown in mMRS-Tween80 or -Tween 20 was analyzed (**Figure 5**). The GP values decreased with increasing ethanol concentration, indicating that ethanol increased the fluidity of the membrane. The response of the membrane fluidity of *L. plantarum* TMW1.460 and TMW1.460Δ*lah* to ethanol was similar. Tween 80 supplemented medium induced a more fluid membrane in both strains.

### Influence of *lah* on Cell Surface Properties in *L. plantarum*

**FIGURE 6:**
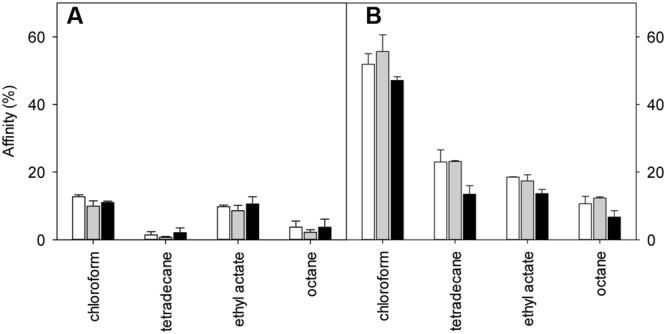
**Effect of 10-lah on cell surface properties of *L. plantarum* grown in different media.** Cell surface hydrophobicity was measured using the MATS method. **(A)** *L. plantarum* TMW1.460; **(B)** *L. plantarum* TMW1.460Δ*lah*. White bar indicates % affinity to solvents when cells were grown in mMRS -Tween 20; gray bar indicated in mMRS (Tween 20) supplemented with 1 g/L oleic acid; black bar indicated in mMRS (Tween 20) supplemented with 1 g/L linoleic acid. Data represent mean ± standard deviation of three independent experiments.

The MATS of *L. plantarum* TMW1.460 and TMW1.460Δ*lah* cultivated in mMRS-Tween20, or mMRS-Tween20 supple mented with oleic or linoleic acid are shown in **Figure 6**. The deletion of *lah* modified the properties of the cell surface. The adhesion of *L. plantarum* TMW1.460Δ*lah* was more solvent-dependent when compared to the wild-type strain and the affinity of cells to the four solvents was generally higher for Δ*lah* mutant than for wild-type strain. Similar trends were noted with strains grown in mMRS with different supplements, suggesting that the differences between wild-type and mutant strains are attributable to 10-LAH, not the product of this enzyme. Similar behavior was observed when strains cultivated in Tween 80 (Data not shown).

In this study, both wild-type and mutant strains exhibited low affinity for tetradecane and octane (non-polar solvents), indicating that the cell surface of both strains was hydrophilic rather than hydrophobic. To determine the effect of deletion of *lah* on the Lewis electron donor/electron acceptor property, the bacterial affinity to chloroform and ethyl acetate between different strains were also compared (**Figure 6**). *L. plantarum* wild-type strain showed similar adhesion ability to chloroform and ethyl acetate. However, regardless of the different supplement in medium, the adhesion of the mutant strain was always higher to chloroform (electron acceptor and acidic solvent) than to ethyl acetate (electron donor and basic solvent).

### Antifungal Properties of Purified Hydroxy Fatty Acids

13-HOE and 10-HOE were purified from organic extracts of cultures of *L. plantarum* TMW1.460Δ*lah* and *L. hammesii*, respectively, when 4 g/L linoleic acid was used as substrate. The major impurity detected in the organic phase was linoleic acid; other more oxidized forms of hydroxy C_18_ fatty acids were also present. The compounds were purified by silica solid phase microextraction and analyzed by LC-APPI-MS/MS. 10-HOE or 13-HOE were eluted in the 1% isopropanol fraction and a single peak was observed in the LC-APPI-MS/MS chromatogram after purification. The antifungal activity of purified 13-HOE and 10-HOE against *A. niger* and *P. roqueforti* is shown in **Table 3**, and compared to reference lipids differing in the number and position of hydroxyl groups or double bonds. Both coriolic acid and ricinoleic acid were active against all fungi indicators with MICs between 0.26 and 0.29. Linoleic acid showed the lowest antifungal activity.

**Table 3:** Minimum inhibitory concentrations (MICs) of hydroxy fatty acids extracted from cultures of *L. hammesii* and *L. plantarum* TMA1.460Δ*lah* and reference fatty acids (*n* = 3).

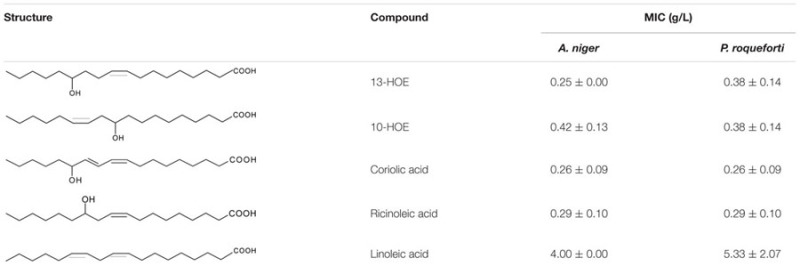

## Discussion

Linoleate hydratases are highly conserved in both Gram-positive and Gram-negative bacteria (Volkov et al., 2010). Our study revealed that 10-LAH and 13-LAH, or enzymes that produce both 10-HOE and 13-HOE are not distinguished by phylogenetic and sequence analysis. All hydratases from lactobacilli examined in this study were determined as 10-LAH, however, they are distributed in two different clusters which also contain 13-LAH. Moreover, the topology of the protein tree disagreed with the evolutionary relationship of the organisms (Zheng et al., 2015), indicating that LAHs are accessory proteins (Wyllie et al., 2000).

Linoleate 10-hydratase is a FAD-containing enzyme and exhibits flavin-like UV absorbance (Rosberg-Cody et al., 2011). FAD cofactor is bound to the conserved FAD binding motif of 10-LAH and stabilizes the active conformation of the enzyme but it is not directly involved in catalysis (Volkov et al., 2010; Yang et al., 2013). The non-covalently bound FAD is easily lost in the purification process (Volkov et al., 2013). This study confirmed that 10-LAH is a FAD-dependent enzyme (Joo et al., 2012) and demonstrated that purification of active 10-LAH from inclusion bodies required addition of FAD. Different from the LAH of *S. pyogenes*, which catalyzes formation of 10-HOE and 13-HOE (Volkov et al., 2010), the 10-LAH characterized in this study formed exclusively 10-HOE from linoleate. The comparison of products formed by *L. plantarum* TMS1.460, the 10-LAH deficient mutant of this strain, and the 10-LAH of this strain strongly suggest that 13-HOE and 10,13 dihydroxy octadecanoic acid formation by this strain is attributable to a linoleate 13-hydratase that was recently characterized in *L. acidophilus* (Park et al., 2015).

Unsaturated fatty acids are essential for growth of many LAB (Johnsson et al., 1995; Gänzle et al., 2000) but high concentrations may inhibit growth of LAB (Guerrini et al., 2002). In contrast, in the present work oleic and linoleic acids stimulated growth of *L. reuteri, L. hammesii*, and *L. sanfranciscensis*. The bacteriostatic and bactericidal activities exerted by UFAs against lactobacilli is strain dependent (Jenkins and Courtney, 2003); the observation that the LAH-negative *L. sanfranciscensis* was the only strain that was inhibited by oleic and linoleic acids suggests that LAH contributes to these strain-specific differences.

Oleic acid modulated membrane fluidity, and influenced the ethanol tolerance of *L. plantarum* TMW1.460 and TMW1.460Δ*lah*. Consistent with our results, addition of Tween 80 to the growth medium increased viability of *Oenococcus oeni* in wine (Guerrini et al., 2002) and supplementation of UFAs to *Saccharomyces cerevisiae* protected against stress (Mishra and Prasad, 1989). However, we observed no difference in ethanol resistance between *L. plantarum* TMW1.460 and TMW1.460Δ*lah.* The protective effect of 10-LAH that was previously observed in exponential phase bifidobacteria may relate to the presence of the enzyme rather than its products (Rosberg-Cody et al., 2011; O’Connell et al., 2013).

The physiochemical properties of cell surface play a critical role in adhesion of pathogens and probiotics to intestinal surfaces. An evaluation of surface properties is achieved by determination of MATS (Bellon-Fontaine et al., 1996; Rosenberg, 2006). Both *L. plantarum* wild-type and *lah* mutant strains displayed a hydrophilic surface character with weak adhesion to non-polar solvents. Similar results were obtained in other *Lactobacillus* spp. and *Lactococcus* spp. (Pelletier et al., 1997; Boonaert and Rouxhet, 2000; Ly et al., 2006). The hydrophobicity of the cell surface changed when the bacteria were cultivated in medium with addition of (UFAs; Kankaanpää et al., 2004; Muller et al., 2011), which was not observed in our study. Bacteria grown in mMRS medium with or without different supplement exhibited similar affinity to solvents. However, *lah* deficiency resulted in a fundamental change in the profile of solvent affinity. Compared with wild-type strain, *L. plantarum* TMW1.460Δ*lah* presented more basic and electron-donating properties. The bacteria with basic character are considered to possess COO^-^ and HSO_3_^-^ chemical groups on their cell surface (Pelletier et al., 1997). *Lactobacillus casei* BL83, BL208 and BL229 also displayed low cell adhesion that was associated with their basic surface property (Muñoz-Provencio et al., 2009). Indeed, the deletion of 10-LAH was involved in the reduced adherence to human keratinocytes by *S. pyogenes* and to human intestinal epithelial cells by *L. acidophilus* (O’Flaherty and Klaenhammer, 2010; Volkov et al., 2010). In our study, 10-LAH mediated differences in cell surface properties may explain the changed cell adhesion to human cells in the previous reports.

Linoleic acid possesses antifungal activity against the plant pathogenic fungi, especially for *Crinipellis perniciosa* at the concentration of 100 μM (Walters et al., 2004). In our study, linoleic acid was less active against *A. niger* and *P. roqueforti*, fungi that are commonly found in cereals and cereal products (Legan, 1993; Sjögren et al., 2003). Remarkably, the MIC of 13-HOE was approximately 15 times lower than that of linoleic acid. Therefore, 13-HOE may be as suitable as 10-HOE as an antifungal agent in foods (Black et al., 2013b). The antifungal activity of hydroxy fatty acids is likely related to their partitioning into lipid bilayers, thus increasing membrane permeability (Sjögren et al., 2003). The MICs of 13-HOE, 10-HOE along with coriolic acid and ricinoleic acid were all comparable, suggesting that all unsaturated monohydroxy fatty acids of C_18_ varying in their hydroxyl group position or degree of unsaturation exert antifungal property. Similar inhibition activities of *Aspergillus* and *Penicillium* spp. were detected among 9- and 13-hydroxy of C_18_ UFA analogs of plant oxylipins (Prost et al., 2005). In contrast, dihydroxy saturated C18 fatty acids do not display antifungal activity (Black et al., 2013b).

## Conclusion

Our study revealed that the differentiation of accessory proteins between 10-LAH and 13-LAH cannot be achieved by phylogenetic analysis. Thus the LAHs from lactobacilli were characterized by heterologous expression and identified as FAD-dependent 10-LAH. Generation of a 10-LAH deficient mutant of *L. plantarum* demonstrated that 13-HOE generated by a different and dedicated hydratase, and is a novel antifungal hydroxy fatty acid. The most prominent physiological difference of the 10-LAH deficient mutant and the wild-type strain was the altered surface hydrophobicity of the bacterial cells. *L. plantarum* is part of the phyllosphere of many plants (Minervini et al., 2015) and oxylipids are an important component of the plant defense against pathogens (Prost et al., 2005). It is possible that the lipid-converting properties of LAHs and their influence on cell surface properties are components of host–microbe interactions.

## Author Contributions

YC and NL conducted experiments, YC and MG wrote the manuscript, JC contributed to experimental design and writing of the manuscript.

## Conflict of Interest Statement

The authors declare that the research was conducted in the absence of any commercial or financial relationships that could be construed as a potential conflict of interest.

## Footnotes

1 http://www.ncbi.nlm.nih.gov
